# Efficacy and Safety of Intravenous Thrombolysis in Patients with Unknown Onset Stroke: A Meta-Analysis

**DOI:** 10.1155/2019/5406923

**Published:** 2019-09-03

**Authors:** Di Luan, Yuanxiang Zhang, Qian Yang, Zhiming Zhou, Xianjun Huang, Shoucai Zhao, Lili Yuan

**Affiliations:** ^1^Department of Neurology, The First Affiliated Hospital of Wannan Medical College, Wuhu, Anhui Province, China; ^2^Department of Clinical Pharmacy, The First Affiliated Hospital of Wannan Medical College, Wuhu, Anhui Province, China

## Abstract

**Objectives:**

Unknown onset stroke (UOS) is usually excluded from intravenous thrombolysis concerning the unclear symptom onset time. Attempts have been done to use thrombolytic therapy in these patients. The current meta-analysis was done to examine the efficacy and safety of intravenous thrombolysis in UOS.

**Methods:**

PubMed, Web of Science, and Cochrane Library were searched for studies comparing thrombolysis with conservative therapy among UOSs. Data of good outcome (mRS, 0-2), mortality, and intracerebral hemorrhage (ICH) and symptomatic ICH (sICH) were extracted and analyzed using the Revman 5.2 software.

**Results:**

In total, 8 studies with 1271 subjects (542 with thrombolysis and 729 with conservative therapy) were included in this meta-analysis. The data showed that patients receiving thrombolysis had a higher incidence of 90-day good outcome (*P* = 0.0005) than conservative therapy. The comparison of discharge (*P* = 0.89) and 90-day mortality (*P* = 0.10) in both groups did not find any significances. The incidences of ICH (*P* = 0.42) and sICH (*P* = 0.06) were relatively comparable between the two therapies.

**Conclusions:**

Intravenous thrombolysis is a better choice for UOS patients for its efficacy and safety. In addition, pretreatment imaging assessment is beneficial for improving the efficacy of thrombolytic therapy. However, it needs more supporting evidences for clinical use in the future.

## 1. Introduction

Ischemic stroke is one of the most common causes of death globally [1, 2]. Intravenous tissue plasminogen activator (rtPA) is recommended for acute ischemic stroke within the time window [3, 4]. It is proven to be effective to save neurological functions against stroke in clinical practice, and it has become a keystone of acute stroke treatment [5, 6].

However, in a certain proportion of stroke sufferers, the clear time point of symptom onset cannot be known. Patients with unknown onset stroke (UOS) may wake up with stroke (WUS) symptoms or cannot state the exact time for unconsciousness [7]. This kind of stroke then poses a challenge for neurological physicians to make appropriate therapeutic decisions for these patients. The unclear symptom onset time may lead to the exclusion of many patients from the first-line thrombolytic therapy. However, efforts have been done to investigate the clinical features and the possibility to apply thrombolytic agents in these patients. Aoki et al. [8] and Schwamm et al. [9] found that thrombolytic therapy was safe and effective in patients with UOS who had diffusion-weighted imaging (DWI)/fluid-attenuated inversion recovery (FLAIR) mismatch. Some studies indicated the similar imaging and clinical characteristics between UOS and stroke with known onset time [10]. And intravenous rtPA may still be beneficial for these patients [11, 12]. But there is still a controversy facing the therapeutic selection for this kind of stroke. Therefore, we conduct this meta-analysis to summarize the current evidences in this field.

## 2. Methods

### 2.1. Search Strategy

This meta-analysis was conducted according to the Preferred Reporting Items for Systematic Reviews and Meta-analysis (PRISMA) [13] and Meta-analysis of Observational Studies in Epidemiology (MOOSE) [14] recommendations. PubMed, Web of Science, and Cochrane Library were searched up to September 1, 2018, using the following terms with various combinations: (i) unknown onset stroke or unclear onset stroke or UOS or wake-up stroke or WUS and (ii) thrombolysis or thrombolyticor fibrinolysis or urokinase or alteplase or rt-PA or rtPA or t-PA or tPA. The articles yielded were then analyzed by two independent researchers for potential studies comparing intravenous thrombolysis with conservative therapy in UOS patients. Studies without clear description of patient characteristics or treatment details or outcomes were excluded. Since the study was done based on the published articles, no ethical approval and patient consent were needed.

### 2.2. Data Extraction

Data in each study were extracted by two authors independently. Any disagreement was resolved by consensus-based discussion among the authors and determined by the senior author. UOS means strokes with discordant last-known normal time and first-found abnormal time [15, 16]. Data, including authors, publication year, trial design, study period, patient number, age, gender, disease history, Toast classification [17], time parameters, neuroimaging methods, National Institutes of Health Stroke Scale (NIHSS) [18], modified Rankin Scale (mRS), and the incidence of intracerebral hemorrhage (ICH) and symptomatic ICH (sICH), were included. For imaging details, technics applied in each study were collected, including noncontrast CT, CT perfusion, CT angiography, and magnetic resonance imaging (MRI) sequences. Time parameters included time from last seen normal (LSN) to symptom onset, LSN to door, LSN to thrombolysis, symptom to door, symptom onset to thrombolysis, and door to treatment. Treatment efficacy was measured at two levels: discharge and 90-day post charge, defined as good outcome (mRS 0-2) and mortality [19]. Therapy safety was assessed using the development of ICH and sICH. sICH was defined as any ICH detected on noncontrast computed tomography (CT) associated with a greater than or equal to a 4-point increase in NIHSS within 48 hours after treatment [20, 21].

### 2.3. Quality Assessment, Sensitivity Analysis, and Publication Bias Assessment

The quality assessment of observational studies was done according to the Newcastle-Ottawa Quality Assessment Scale [22, 23] in terms of patient selection, comparability of the study groups, and assessment of outcome. A score of 0–9 was used for each study. Studies that achieved six or more stars were considered to be of high quality. The Cochrane Risk of Bias Tool [24, 25] was adopted to explore the risk of bias for each randomized controlled trial (RCT). The following items were analyzed: generation of allocation sequence, allocation concealment, blinding (participants and personnel), blinding (outcome assessment), incomplete outcome data, selective reporting, and other sources of bias. Sensitivity analysis was done using the leave-one-out method to test the stability of the results and the source of heterogeneity if necessary. Publication bias analysis was done with funnel plots if the number of included studies exceeded 10.

### 2.4. Statistical Analysis

The meta-analysis was done using the Revman 5.2 software. The odds ratio (OR) was used to compare dichotomous variables. All results were displayed with 95% confidence intervals (CIs). Heterogeneity was quantified by the estimated *I*^2^ with a Cochrane *Q* test. When the level of *I*^2^ was ≥50% or *P* ≤ 0.10, the results were considered by the application of the random effects model. Otherwise, it was considered using the fixed effects model.

## 3. Results

### 3.1. Study Search and Study Characteristics

**Figure 1:**
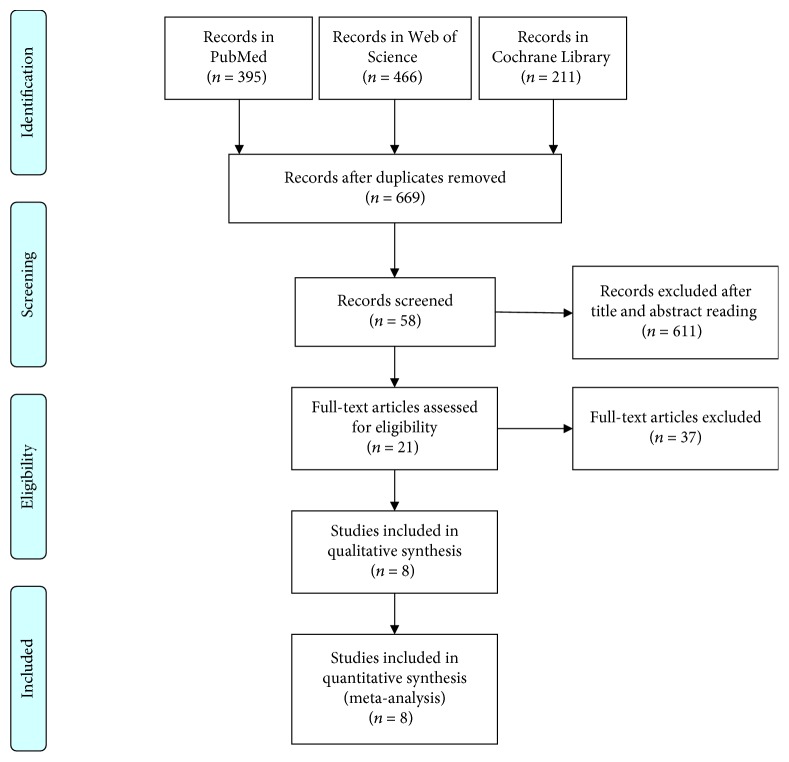
Flow diagram of the study inclusion process in this meta-analysis.

The search diagram was shown in Figure 1. A systematic search in PubMed, Web of Science, and Cochrane Library yielded 1072 articles. Then 403 duplicates were removed. After screening by going through titles, abstracts, and whole texts, there were 8 studies [12, 15, 16, 25–29] with 1271 subjects (542 with thrombolysis and 729 with conservative therapy) after screening (Table 1). Six countries contributed to the production of the studies: USA [15, 16], Germany [12, 28], Switzerland [29], Korea [16], UK [27], and Canada [25]. Among them, 3 were retrospective studies [15, 25, 26], 3 were prospective studies [16, 27, 28], and 2 were RCTs [12, 29]. The Toast classification was available in 7 studies [15, 16, 25–27, 29], and one single study only enrolled those patients with proximal large artery occlusion [25]. All the studies used rtPA as the thrombolytic agent.

One single study [26] only used noncontrasted CT as the diagnostic method, 2 used MRI-based approach for pretreatment screening [12, 16], 3 studies applied CT-based method for screening [25, 27, 29], and 2 studies used MRI- plus CT-based technics [15, 28]. All studies indicated 90-day mRS, except 1 study only indicating the scale at discharge level [26]. All non-RCTs were with relatively high quality (Table 1). Two RCTs kept a good control in each domain (Table 2).

### 3.2. Outcome Assessment

**Figure 2:**
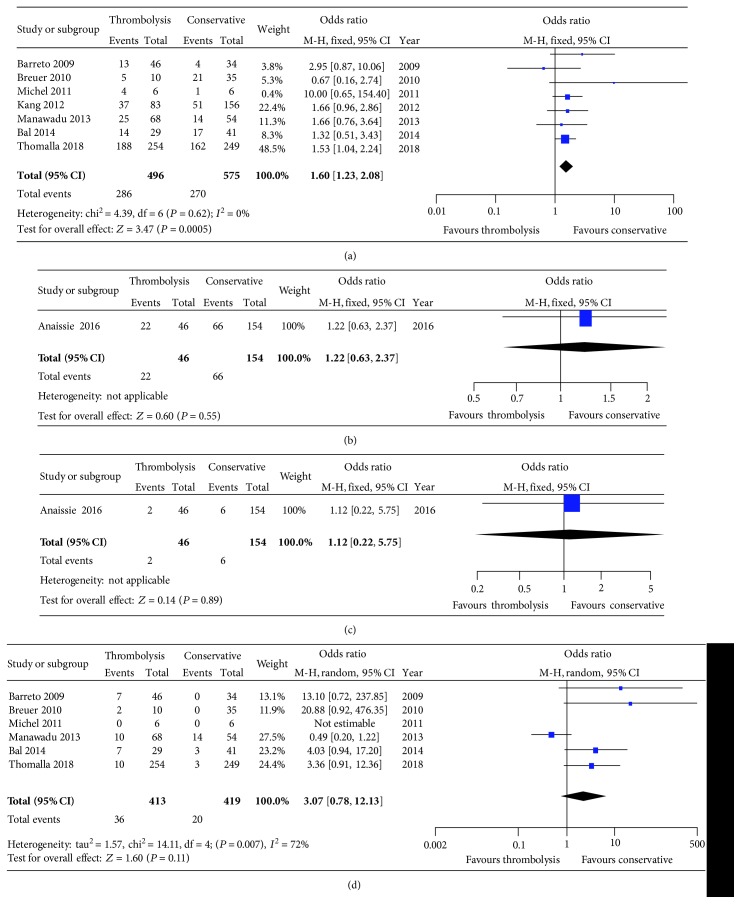
Forest plots of good outcome and mortality between thrombolysis and conservative therapy. (a) 90-day good outcome; (b) discharge good outcome; (c) discharge mortality; (d) 90-day mortality.

All the outcomes were shown in Table 3. The pooling of good outcome showed that patients receiving thrombolysis intended to have a higher rate in 90 days than conservative therapy after treatment (57.66% *vs.* 46.96%; *P* = 0.0005) (Figure 2(a)). But we did not found any differences with respect to discharge good outcome (47.82% *vs.* 42.85%; *P* = 0.55) (Figure 2(b)). And the comparison of discharge (4.35% *vs.* 3.90%; *P* = 0.89) (Figure 2(c)) and 90-day mortality (8.72% *vs.* 4.77%; *P* = 0.11) (Figure 2(d)) did not find any significances.

**Figure 3:**
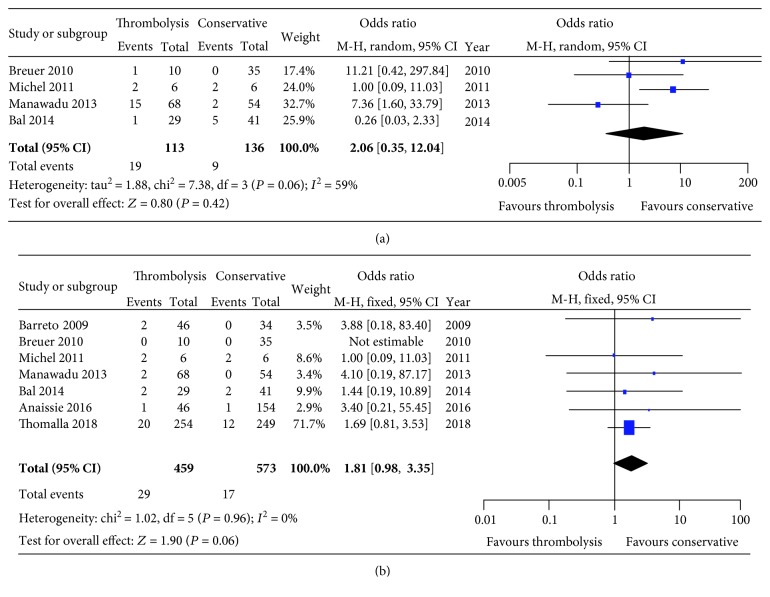
Forest plots of ICH (a) and sICH (b) incidences between thrombolysis and conservative therapy. ICH: intracerebral hemorrhage; sICH: symptomatic intracerebral hemorrhage.

For safety analysis, the incidences of ICH (16.81% *vs.* 6.62%; *P* = 0.42) (Figure 3(a)) and sICH (6.32% *vs.* 2.97%; *P* = 0.06) (Figure 3(b)) were relatively comparable between the thrombolysis and conservative therapy.

We also did further analysis based on imaging methods (Table 3). In studies using MRI, a higher incidence of 90-day good outcome (*P* = 0.005) was seen in the thrombolysis group. And no changes of trends in 90-day mortality and sICH were seen. When analyzing CT-based approaches in 3 studies [25, 27, 29], no favorable results were seen in each outcome. Two studies used CT and MRI methods in preexamination. And no differences between the two groups in the outcomes of 90-day good outcome, ICH, and sICH were revealed, except a lower rate of 90-day mortality in conservative therapy (*P* = 0.01).

### 3.3. Heterogeneity, Sensitivity Analysis, and Publication Bias Assessment

There were relatively high heterogeneity in 90-day mortality (*I*^2^ = 74%) and ICH incidence (*I*^2^ = 59%). We then used the leave-one-out method to analyze these results. The results of 90-day mortality turned significant (*P* = 0.0003) with a robust change of heterogeneity (*I*^2^ = 0%) when the study by Manawadu et al. [27] was extracted. Also, without the study by Bal et al. [25], ICH incidence is relatively higher in the thrombolysis group than in the conservative therapy group (*P* = 0.0006, *I*^2^ = 9%). As there were less than 10 studies in this meta-analysis, the publication bias assessment cannot be performed accurately.

## 4. Discussion

The current study is aimed at comparing intravenous thrombolysis with conservative therapy in UOS patients. We found that intravenous thrombolysis induced a higher incidence of 90-day good outcome without increased mortality, compared with conservative therapy.

UOS patients are unable to provide accurate symptom onset time for some reasons, for example, awakening with stroke symptoms, nonwitnessed stroke with aphasia or unconsciousness [30, 31]. They are usually considered as a contraindication for intravenous thrombolysis for the uncertain symptom onset. There is an increasing attention to determine the potential role of thrombolytic therapy for patients in this situation [32–34]. A trial in 2009 [15] indicated that thrombolysis-treated WUS had higher rates of excellent and favorable outcome but higher mortality than those receiving conservative treatment. But some studies pointed that thrombolysis may be as safe as conservative therapy in WUS [25], with an even better outcome [26]. The present analysis found a higher 90-day good outcome rate in thrombolysis than conservatives, pointing out that intravenous thrombolysis should be beneficial for UOS. Although it indicated comparable effects between the two therapies at discharge level, only one single study [26] was included in the outcome. More trials should be done at this time point. And the mortality in both groups did not differ in the short and long term, implying the comparable safety between thrombolysis and conservative therapy. ICH is a severe situation in acute ischemic stroke following thrombolytic agent use [35]. It is also confirmed in this meta-analysis that ICH and sICH incidences were similar in patients receiving the two therapies, ensuring again the safety of thrombolysis.

The pretreatment neuroimaging evaluation is becoming an important factor for UOS. Noncontrast CT is the common method for stroke patients after initial admission. CT perfusion and MRI are methods with increasing application for screening, which have unique roles for patient selection. The mismatch in noncontrast CT with CT perfusion and MRI indicated that some UOS patients are eligible for intravenous thrombolysis [16]. The inconsistent use of neuroimaging methods was seen in the current study. Here, we did subgroup analysis on this basis. Only one study used noncontrast CT for neuroimaging [26]. Two studies selected patients for thrombolysis according to the mismatch between DWI and FLAIR. Positive DWI and negative FLAIR changes identify stroke within 4.5 hours [12]. Obviously, patients using MRI for screening had an increased incidence with 90-day good outcome. Some researchers believe that CT perfusion and CT angiography are also able to define suitable patients for rtPA use [25, 27]. However, the pooling of data from CT perfusion or CT angiography did not indicate the potential benefits of thrombolysis in recovery. Also, data with mixed use of MRI and CT perfusion or CT angiography also did not reveal any differences in 90-day good outcome. Then, it seems that MRI should be a better choice for patient selection into thrombolytic treatment. But the fact that there is a lack of MRI in some small centers and the long duration of imaging may impair the wide use of MRI in the clinical practice. The inconsistent use of neuroimaging methods may hinder the reliability of the results. And studies in these comparisons were limited, calling for more trials.

A higher heterogeneity was found in the results of 90-day mortality and ICH. And the sensitivity analysis demonstrated the stable results of 90-day good outcome and sICH. But 90-day mortality turned significant (*P* = 0.0003) with a robust reduction of heterogeneity from 75% to 0% by excluding the study by Manawadu et al. [27]. We found that not all the subjects in this study received CTP in the inclusion process. Some patients may be not suitable for thrombolysis, but they still received thrombolysis. Then, a higher rate of 90-day mortality (7.54%) than conservative therapy (1.37%) may also explain it. Without the study by Bal et al. [25], ICH incidence is relatively higher in the thrombolysis group than in the conservative therapy group (*P* = 0.0006, *I*^2^ = 9%). The review of this article found a higher hemorrhagic infarction in the conservative group. It is believed that hemorrhagic infarction is likely to induce an occurrence of ICH [36]. Also, the admission time from symptom onset was not available. These should demonstrate the high heterogeneity caused by the study.

Two clinical trials, EXTEND [37] and THAWS [38], are aimed at exploring the safety and efficacy of rtPA in the treatment of UOS and WUS patients and may provide additional evidence.

Tenecteplase, a third-generation thrombolytic, compared with rtPA, has a stronger binding ability to fibrin and is more resistant to the inactivation of plasminogen activator inhibitor-1 [39]. Single doses may be administered rather than sustained administration due to longer half-life. In the treatment of myocardial infarction, tenecteplase is equivalent to rtPA, but the risk of bleeding is reduced [40]. Burgos and Saver [41] found in a meta-analysis involving 5 RCTs that tenecteplase is not inferior to rtPA in the treatment of acute ischemic stroke. Kheiri et al. [42] conducted a network meta-analysis and found that tenecteplase had a higher recanalization rate and more favorable early neurological function improvement than rtPA in the treatment of acute ischemic stroke, and there was no difference in safety. Based on the above, whether tenecteplase is safer and more effective in the treatment of UOS needs to be further studied.

There were some limitations in this meta-analysis. First, most of the studies included were observational studies, in which intravenous rtPA was given based on clinical decision and imaging assessment. This lays a potential bias in the analysis. Enrollment of RCTs can help solve this, but only 2 studies were found in this study. Also, it encouraged high-quality large-sample RCTs focusing on this point to evaluate the role of intravenous rtPA.

And the number of the included studies was limited. This made it difficult to assess publication bias accurately. Also, the sample size in each study was small and they came from different regions. Moreover, the preexamination imaging methods were not consistent in all the studies, which indicated the variety in patient enrollment. And the high heterogeneity meant the unstable results of certain variables. This hindered the credibility of our results.

Intravenous thrombolysis is a better choice for UOS patients for its efficacy and safety. And pretreatment MRI assessment is beneficial for improving the efficacy of thrombolytic therapy. However, it needs more supporting evidences for clinical use in the future.

## Figures and Tables

**Table 1:** The baseline characteristics of the included studies in the meta-analysis (study quality of non-RCTs was shown).

Study	Barreto 2009 [15]	Breuer 2010 [28]	Michel 2011	Kang 2012 [16]	Manawadu 2013 [27]	Bal 2014 [25]	Anaissie 2016 [26]	Thomalla 2018 [12]
*Region*	USA	Germany	USA	Korea	UK	Canada	USA	Germany
*Design*	Retrospective	Prospective	RCT	Prospective	Prospective	Retrospective	Retrospective	RCT
*Period*	2003.03-2008.01	2006.10-2008.05	2004.06-2007.12	2006.09-2009.07	2009.01-2010.12	2003.01-2010.03	2008.07-2014.05	2012.09-2017.06
*Outcome assessment time*	NA	90 days	90 days	90 days	90 days	90 days	90 days	90 days
*Admission time (h)*	3	6	3	3	4.5	NA	4.5 h	4.5 h
*Preexamination*	All NCT, 10 CT perfusion, 6 MRI	All NCT, 43 MRI, 2 CTA	All NCT+CT perfusion	All NCT+MRI	All NCT, 64 CTP	All NCT, CTA	All NCT	All NCT+MRI
*No. of patients*								
Thrombolysis	46	10	6	83	68	29	46	254
Conservative	34	35	6	156	54	41	154	249
*Age (y)*								
Thrombolysis	62 ± 14^a^	73 (57-92)^b^	69.5 (57-78)^b^	NA	73.9 ± 15.6^a^	68 (23)^c^	69 (42-98)^b^	65.3 ± 11.2^a^
Conservative	64 ± 13^a^	66 (39-87)^b^	49 (44-78)^b^	NA	70.6 ± 16.7^a^	74 (21)^c^	63 (22-93)^b^	65.2 ± 11.9^a^
*Gender*								
Thrombolysis	39	5	3	55	23	12	22	165
Conservative	44	23	3	88	28	17	85	160
*NIHSS*								
Thrombolysis	16 (3-24)^b^	10.5 (1-22)^b^	17 (13-21)^b^	14 (10-18)^c^	11.5 (8-17)^c^	14 (11)^c^	9.5 (1-27)^b^	6 (4–9)^c^
Conservative	10.5 (2-26)^b^	6 (1-21)^b^	14.5 (12-19)^b^	12 (6.25-17)^c^	9 (5-15)^c^	13 (11)^c^	5 (0-33)^b^	6 (4–9)^c^
*HT*								
Thrombolysis	30	9	4	54	45	18	36	135
Conservative	22	29	1	94	32	27	119	131
*DM*								
Thrombolysis	10	4	1	23	14	3	20	43
Conservative	11	13	0	57	13	7	45	39
*CAD*								
Thrombolysis	8	3	0	NA	NA	NA	NA	NA
Conservative	5	6	0	NA	NA	NA	NA	NA
*Smoking*								
Thrombolysis	NA	1	0	30	8	13	NA	NA
Conservative	NA	9	2	54	7	19	NA	NA
*AF*								
Thrombolysis	NA	NA	2	NA	21	10	6	30
Conservative	NA	NA	0	NA	9	18	21	29
*Previous stroke*								
Thrombolysis	NA	5	NA	19	NA	NA	NA	37
Conservative	NA	9	NA	31	NA	NA	NA	31
*Hyperlipidemia*								
Thrombolysis	12	8	4	24	22	9	19	93
Conservative	9	27	3	40	25	6	60	85
*TOAST classification*								
*Cardioembolic*								
Thrombolysis	20	4	2	31	33	NA	11	NA
Conservative	14	8	0	57	21	NA	34	NA
*Large artery atherosclerosis*								
Thrombolysis	15	2	2	46	14	NA	8	NA
Conservative	7	10	0	77	11	NA	27	NA
*Small vessel*								
Thrombolysis	1	1	0	0	4	NA	3	NA
Conservative	5	7	4	0	12	NA	53	NA
*Unknown*								
Thrombolysis	5	2	2	NA	13	NA	17	NA
Conservative	5	8	2	NA	13	NA	25	NA
*Other*								
Thrombolysis	5	1	0	6	4	NA	7	NA
Conservative	2	2	0	22	2	NA	14	NA
*LSN to symptom onset*								
Thrombolysis	NA	NA	NA	NA	NA	NA	NA	7.2 (4.7–8.7) h^c^
Conservative	NA	NA	NA	NA	NA	NA	NA	7.0 (5.0–9.0) h^c^
*LSN to door*								
Thrombolysis	NA	508 (200-691) min^b^	NA	8.6 (5.4-11.1) h^c^	NA	NA	NA	NA
Conservative	NA	577 (182-849) min^b^	NA	7.8 (4.9-11.7) h^c^	NA	NA	NA	NA
*LSN to thrombolysis*								
Thrombolysis	10.7 ± 4.3 h^a^	NA	564 (390–805) min^b^	NA	NA	NA	NA	10.3 (8.1–12.0) h^c^
Conservative	NA	NA	437.5 (330–656) min^b^	NA	NA	NA	NA	10.4 (8.1–12.1) h^c^
*Symptom onset to door*								
Thrombolysis	2.0 ± 1.9 h^a^	93 (20-287) min^b^	NA	1.7 (0.9-2.7) h^c^	NA	526 ± 112 min^a^	NA	2.6 (1.9–3.3) h^c^
Conservative	3.7 ± 3.6 h^a^	95 (38-360) min^b^	NA	2.0 (1.0-3.6) h^c^	NA	540 ± 140 min^a^	NA	2.6 (2.1–3.3) h^c^
*Symptom onset to thrombolysis*								
Thrombolysis	4.3 ± 3.3 h^a^	NA	NA	4.6 (2.8-6.0) h^c^	NA	516 ± 160 min^a^	NA	3.1 (2.5–3.8) h^c^
Conservative	NA	NA	NA	NA	NA	NA	NA	3.2 (2.6–3.9) h^c^
*Door to treatment*								
Thrombolysis	2.4 ± 1.9 h^a^	80 (45–127) min^b^	109.5 (85–131) min^b^	155 (100–195) min^c^	NA	NA	NA	25 (16–35) min^c^
Conservative	NA	NA	113 (75–205) min^b^	NA	NA	NA	NA	26 (18–37) min^c^
*Good outcome*								
Thrombolysis	13	5	4	37	25	14	22	188
Conservative	4	21	1	51	14	17	66	162
*Mortality*								
Thrombolysis	7	2	0	NA	10	7	2	10
Conservative	0	0	0	NA	14	3	6	3
*NIHSS at 24 h*								
Thrombolysis	NA	7.5 (0-18)^b^	10.5 (7-19)^b^	NA	6 (2, 13.5)^c^	10 (13)^c^	4 (0-24)^b^	NA
Conservative	NA	5 (0-21)^b^	19.5 (6-24)^b^	NA	5 (3, 10)^c^	10.5 (15.5)^c^	3 (0-29)^b^	NA
*NIHSS change at 24 h*								
Thrombolysis	NA	NA	NA	NA	-4 (-8, 0)^c^	NA	-2 (-16, 19)^b^	NA
Conservative	NA	NA	NA	NA	-3 (-4, 0)^c^	NA	-1 (-13, 17)^b^	NA
*NIHSS at discharge*								
Thrombolysis	NA	6 (0–21)^b^	NA	NA	NA	NA	3 (2-42)^b^	NA
Conservative	NA	3 (0–15)^b^	NA	NA	NA	NA	3 (0-42)^b^	NA
*sICH*								
Thrombolysis	2	0	2	NA	2	2	1	20
Conservative	0	0	2	NA	0	2	1	12
*ICH*								
Thrombolysis	NA	1	2	NA	15	5	NA	NA
Conservative	NA	0	2	NA	2	1	NA	NA
*Study quality*	7	6	NA	8	8	7	6	NA

RCT: randomized controlled trial; CT: computed tomography; NCT: noncontrast CT; MRI: magnetic resonance imaging; a: mean ± SD; b: median (minimum-maximum); c: mean (interquartile); y: year; h: hour; min: minute; NA: not applicable; HT: hypertension; DM: diabetes mellitus; CAD: cardiovascular disease; AF: arterial fibrillation; mRS: modified Rankin Scale; NIHSS: National Institutes of Health Stroke Scale; LSN: last seen normal; ICH: intracerebral hemorrhage; sICH: symptomatic ICH.

**Table 2:** Quality assessment of RCT in this meta-analysis. RCT: randomized controlled trial.

Study	Random sequence generation	Allocation concealment	Blinding (participants and personnel)	Blinding (outcome assessment)	Incomplete outcome data	Selective reporting	Other sources of bias
Michel	Low	Low	Low	Low	Low	Low	Low
Thomalla	Low	Low	Low	Low	Low	Low	Low

**Table 3:** Overall and subgroup meta-analysis of the included studies.

				Study heterogeneity			
Outcomes	No.	OR [95% CI]	*P*	*χ* ^2^	df	*I* ^2^ (%)	*P*-*Q* test
*Overall analysis*							
90-day good outcome	7	1.60 [1.23, 2.08]	0.0005	4.39	6	0	0.62
Discharge good outcome	1	1.22 [0.63, 2.37]	0.55	NA	NA	NA	NA
90-day mortality	6	3.07 [0.78, 12.13]	0.11	14.11	4	72	0.007
By discharge mortality	1	1.12 [0.22, 5.75]	0.89	NA	NA	NA	NA
ICH	4	2.06 [0.35, 12.04]	0.42	7.38	3	59	0.06
sICH	7	1.73 [0.98, 3.05]	0.06	1.29	5	0	0.94
*Subgroup analysis*							
*MRI*							
90-day good outcome	2	1.57 [1.15, 2.15]	0.005	0.05	1	0	0.82
90-day mortality	1	3.36 [0.91, 12.36]	0.07	NA	NA	NA	NA
sICH	1	1.63 [0.82, 3.27]	0.17	NA	NA	NA	NA
*CT*							
90-day good outcome	3	1.68 [0.93, 3.01]	0.08	1.88	2	0	0.39
90-day mortality	3	1.30 [0.17, 10.18]	0.8	5.82	2	83	0.02
ICH	3	1.41 [0.17, 11.49]	0.75	6.46	2	69	0.04
sICH	3	1.56 [0.50, 4.84]	0.44	0.68	2	0	0.71
*MRI+CT*							
90-day good outcome	2	1.47 [0.34, 6.30]	0.61	2.44	1	59	0.12
90-day mortality	2	16.25 [1.94, 136.20]	0.01	0.05	1	0	0.82
ICH	1	11.21 [0.42, 297.84]	0.15	NA	NA	NA	NA
sICH	2	3.72 [0.18, 75.14]	0.39	NA	NA	NA	NA

OR: odds ratio; *P*: percentage; NA: not applicable; ICH: intracerebral hemorrhage; sICH: symptomatic ICH; MRI: magnetic resonance imaging; CT: computed tomography.

## Data Availability

The data used to support the findings of this study are available from the corresponding author upon request.
